# Comic worksheets for integrated disaster risk reduction learning in the subject of Geography

**DOI:** 10.4102/jamba.v13i1.1149

**Published:** 2021-11-25

**Authors:** Ahmad Yani

**Affiliations:** 1 Department of Geography Education, Faculty of Social Sciences Education, Universitas Pendidikan Indonesia, Bandung, Indonesia

**Keywords:** comics, DRR, Geography, inquiry, learning media, worksheets

## Abstract

Indonesian students have limited visual learning capacity. Low literacy of such students has gained the researcher’s attention in developing their literacy through comic worksheets. The researcher created comic worksheets with blank speech bubbles to help high school students build their conceptual framework about disaster risk mitigation. This study attempts to investigate the effectiveness of comic worksheets in helping high school students learn disaster risk reduction (DRR) in the subject of Geography. This study followed the research and development (R&D) approach to develop comic worksheets with blank speech bubbles. A paired sample *t*-test was used to analyse the mean difference between the pre-and post-test scores of 103 students. The results show a significant difference between both means. It indicates that using comic worksheets could improve student learning outcomes. It could also effectively be used as an assessment instrument for the students’ affective aspects. Dialogues among comic characters compiled by students record their attitudes and behaviour in their everyday life. Collaboration between teachers, learning media developers, and artists is needed to create comic packages for learning Geography.

## Introduction

One of the approaches to include disaster risk reduction (DRR) in school curricula is infusion or permeation within the curriculum of specific school subjects (Selby & Kagawa 2012). In Indonesia, Geography is the most frequently chosen subject since it is a very closely related topic to DRR. However, it has been criticised that it is usually a literal reading of the curriculum where knowledge acquisition becomes the orientation. Therefore, it tends to result in very little infusion. Time constraint is also one of the reasons why DRR topics within the subjects are not covered in-depth, let alone to focus on achieving long-term attitudinal changes (Apronti et al. 2015). However, there are many resources such as various learning media opportunities that can be utilised to change literal learning to a more holistic learning. One such resource that has not been used optimally in various learning areas is student comic worksheets.

It has been successfully proven in many studies that comic worksheets can help teachers overcome the problem of time constraint in learning activities (Lee 2014). However, in Indonesia, comic worksheets only serve as additional tasks or are designed solely to prepare students for the end-of-semester test and national exam. Meanwhile, worksheets in general can be developed to assist students in the inquiry process of learning. Barniol and Zavala (2016) suggested that worksheets should not only contain a collection of practice test questions, but also a systematic guide for the process of scientific activities in building conceptual understanding. Students will not only become adept in understanding the issues, but will also be able to express their opinions in their own words (Kuamr Mishra 2014). To study the value of worksheets, the focus of this study is on comic worksheets.

To support inquiry learning, interesting worksheets are needed. In this case, comic worksheets which contain comic strips are assumed to support inquiry leaning. Some studies on comics used for improving an understanding of disaster mitigation have been conducted. For example, Noviana et al. (2019) showed that comics strips can increase knowledge of disaster mitigation in elementary schools so that students can take appropriate actions before, during, and after a disaster occurs. Suprapto, Mulianingsih and Setyowati (2020) tested the effectiveness of digital comics in increasing students’ understanding of disaster preparedness. Furthermore, Artha, Suryana and Mayar (2020) reported the effectiveness of digital comics (e-comic) in learning disaster mitigation in early childhood education. Syahrul et al. (2019) reported the effect of using comics without text and direct learning on narrative writing skills for grade IV of elementary school students. They indicated in their research that the students’ narrative writing skills could be improved more through learning by using comic strips without texts.

Some relevant studies had been conducted long before those mentioned studies. For example, Hutchinson (1949) had conducted experiments on the effect of comics on learning motivation. Using the comic magazine ‘Puck – The Comic Weekly’, he found that 74% of the teachers stated that comics had motivated learning a lot and 79% of teachers reported that comics could increase student participation (Sharpe & Izadkhah 2014; Sones 1944). Sharpe and Izadkhah (2014) also noted that a fairly influential study on comics was reported to increase the students’ awareness of environmental and health education Worthy, Moorman and Turner (1999) also showed that comic strips are quite popular among children aged 12–13 years. Additionally, Morrison, Bryan and Chilcoat (2002) stated that comics can bridge between student life inside and outside the school.

According to Tate (2015), today, students live in a very visual world. It is predicted that most of the students will be predominately visual. A teacher now tends to use a related picture to support his or her explanation about metamorphosis, hang it on the classroom wall for several days, so that the students can visualise what the picture is. In addition, some more ideas may be put into practice by a teacher in developing worksheets which have the potential to facilitate the students in their inquiry learning.

For the forgone, it can be concluded that comics have proven to be very effective in improving understanding of the subject matter (see Tate 2015). In addition, comics are so interesting and motivating that the messages conveyed through comics can be easily remembered by the students. Series of comics showing the process of disaster mitigation actions are easier to understand than long narratives.

On the other hand, it has become common knowledge that panic in facing the disasters has caused many victims. Many people are not able to use their common sense; they panic and tend to behave irrationally and adopt selfish attitudes (Haghani et al. 2019). They act randomly and follow their instinct to selfishly save themselves by taking advantage of what they still remember. Meanwhile, there have been many studies that state that visual memory is very strong and fundamental in the realm of cognition; the power of visual memory has even not been imitated by artificial intelligence systems (Andreopoulos & Tsotsos 2013; DiCarlo, Zoccolan & Rust 2012; Schurgin 2018). Visualisations stored in memory play an important role in determining how one behaves. It has been reported in psychopathological studies that many patients with post-traumatic stress disorder, depression, and other anxiety disorders often come with recurring visual disturbances. Visual memory usually appears very clear, detailed, and with very popular content (Brewin et al. 2010). For this reason, comics are the right alternative in DRR education for both children and adults. However, to confirm this hypothesis, a study is needed. This research attempts to investigate the effectiveness of comics on learning disaster reduction. The indicators of the effectiveness of comics work about DRR are determined by three things, namely, improving learning outcomes, higher-order thinking and inquiry, and developing students’ affective aspects.

Educational comics contribute to students’ intellectual development and arts. With their attractive appearances, comics can also be entertaining and educative (Maharsi 2011; Russell & Murray 1993; Weitkamp & Burnet 2007). They can increase students’ vocabulary, help them grasp abstract things or formulas, develop their reading and learning interest in a particular field, and the general virtues (Trimo 1997). The researcher has tried to develop worksheets with comic strips with blank speech bubbles to help high school students develop their conceptual framework about DRR. This research employed research and development (R&D) approach to create the worksheets. The study investigated the comic worksheet’s effectiveness for operational field testing and explored the obtained data for improvement in a final product revision.

## Inquiry learning for disaster risk reduction purposes

Disaster risk reduction education aims to build students’ understanding of the causes and effects of hazards, and develop their competencies and skills to contribute proactively to disaster prevention and mitigation. There are several approaches to teach DRR in schools. According to Selby and Kagawa (2012), the infusion or permeation approach is the most frequently used approach. This approach enables the integration of DRR topics into existing subjects. Natural Science and Geography are found to be the most chosen subjects. However, they argue that the infusion of DRR topics in these subjects is limited because both the subjects have cultural assumptions about the learning outcomes, which orient towards acquiring knowledge about natural hazards that are already obsolete, such as tales about disasters which are dominated by myths and fatalism. Apronti et al. (2015) found that another cause for the limited infusion was not enough time allocated in the existing subjects to teach and learn about DRR, especially in terms of prevention, mitigation, and preparedness.

This study followed Selby and Kagawa’s (2012) teaching and learning techniques based on their case studies of DRR education from 30 countries. One of these techniques is inquiry learning. In Geography, this learning approach is used in many education curricula. It covers a range of pedagogical approaches that enable students to do investigative work and develop a critical understanding of earth phenomena (Prince & Felder 2007; Roberts 2013). Investigative learning activities in the form of problem-based, project-based, and case-based methods are often used for DRR education in Geography subjects. For example, Gouramanis and Morales Ramirez (2020) used this approach based on The Sendai Framework for Disaster Risk Reduction (SFDRR) as a pedagogical tool to explore Singaporean undergraduates’ learning of natural hazards in Southeast Asia. They found a significant improvement in students’ knowledge of the physical, human, and economic aspects and interactions before, during, and after a natural hazard. They also emphasised the scaffolded learning-based activities to contribute to good learning outcomes.

Scaffolding can facilitate meaningful learning in students’ inquiry activities (Eysink, Gersen & Gijlers 2015; Minner, Levy & Century 2010). The one used by Gouramanis and Ramirez (2020) is what Saye and Brush (2002) categorised as soft scaffolds, which usually refer to tutorial activities. While on the other hand, there are also what are called hard scaffolds. Students’ worksheets are one of the examples in this category (Belland, Glazewski & Richardson 2008). Literature about the degrees of impacts of students’ worksheets as scaffolds is varied. Some studies demonstrated significant impacts on students’ learning (Barniol & Zavala 2016; Kammerer, Meier & Stahl 2016; Repdayanti & Oktavia 2018; Utami et al. 2016). For example, Kammerer et al. (2016) examined whether a paper-based worksheet intervention effectively supported secondary-school students’ reflection in an Internet-reading task. They provided worksheets with and without source prompts to be filled in by the students to test the effectiveness. The results indicated that the group of students who worked with the worksheets provided source prompts outperforming the other group.

However, some studies have shown students’ worksheets’ ineffectiveness, especially in inquiry learning such as problem-based learning (Choo et al. 2011). Ransom and Manning (2013) also argued that the consistent use of worksheets was counterproductive for student learning and invited educators to think again about whether it can support students’ meaningful learning experiences or not. They explained that the idea of removing worksheets from the classroom had appeared decades ago since Smith (1964) stated that worksheets could not help students to think. Bizar and Daniels (1998) recommended fewer worksheets should be used in the classroom. More than 28 years of Kamii’s continuing research (1980–2008) indicated that using worksheets did not help teachers create meaningful learning experience activities (Kamii 2014).

Although the two seem contradictory, they share the common ground on how the students’ worksheet should be designed and used. Choo et al. (2011) suggested further research for more conclusive findings of worksheets’ effectiveness since it may be varied in design depending on the curriculum to be delivered. Ransom and Manning (2013) noted that not all worksheets are just a piece of paper that contains problems, but some can be useful tools for learning, such as diagrams, maps, or other materials. On the other hand, literature that shows students’ worksheets’ effectiveness also emphasises its design and purposes.

Indeed, worksheets should be designed and developed carefully to meet learning purposes. Instead of being just a ‘sheet’ to be passed out to the class, it has to be a learning media that is attractive and supports students in their inquiry learning experiences. The Internet is a resource full of quality ideas and materials for designing and developing visually appealing students’ worksheets. However, the students’ needs and the condition of their learning environment are also worth considering.

Students in Indonesia have limited visual learning resources, especially the ones accessible to students in rural areas. This problem could probably lead Indonesian students to have low literacy. Based on the PISA 2018 result, the Indonesian students’ reading performance go far below the Organisation for Economic Co-operation and Development (OECD) average in 2018. Out of 79 assessed countries and economies, Indonesia ranked 73rd in mathematics, 74th in reading, and 71st in science (Tehusijarana 2019). Those conditions gained the researcher’s concern for developing worksheets in the form of cartoon-comic. When Constance Kamii has shown the superior effectiveness of games, especially for math instruction, Keogh, Naylor and Wilson (1998) developed a concept cartoon as a teaching and learning strategy in science. Presented characters in the comic strips can relate the topics being studied to students’ daily lives. This enables students to inquire and share their opinions over everyday life problems (Greyling 2012; Keogh & Naylor 1999; Ridha et al. 2019). Keogh and Naylor (1999) investigated the effectiveness of the cartoons via classroom observation in 1998 and collected data by interviewing students and teachers in 1999. They found that that the concept cartoons effectively provided students with a stimulus to construct meaningful explanations and promote students’ investigation activities. These findings were supported by other studies such as Stephenson and Warwick (2002), Kabapınar (2009), Taşlidere (2013). Based on the result of the R&D preliminary study conducted by the researcher, the cartoon-comic was found to be more suitable for students to learn DRR in Geography subject than many different kinds of concept cartoons. However, more studies need to be conducted to explore its effectiveness in various contexts.

## Worksheets containing comics with blank speech bubbles

Comics with blank speech bubbles in this study refer to comics that are not supplemented with dialogues or texts among the characters; instead, some notes are given on the story. The comics consist of four general sections: (1) students’ data; (2) synopsis of the story; (3) 25 scenes as story content; and (4) the questionnaire placed at the end of the comics. The storyline is written in the synopsis part set at the beginning of the comics. The characters’ missing dialogues become part of the students’ tasks, which they will have to complete. Thus, students will feel as if they are directly involved in the comic story. The characters’ speech bubbles contain dialogues regarding knowledge and some others dialogues to assess students’ character values.

**FIGURE 1:**
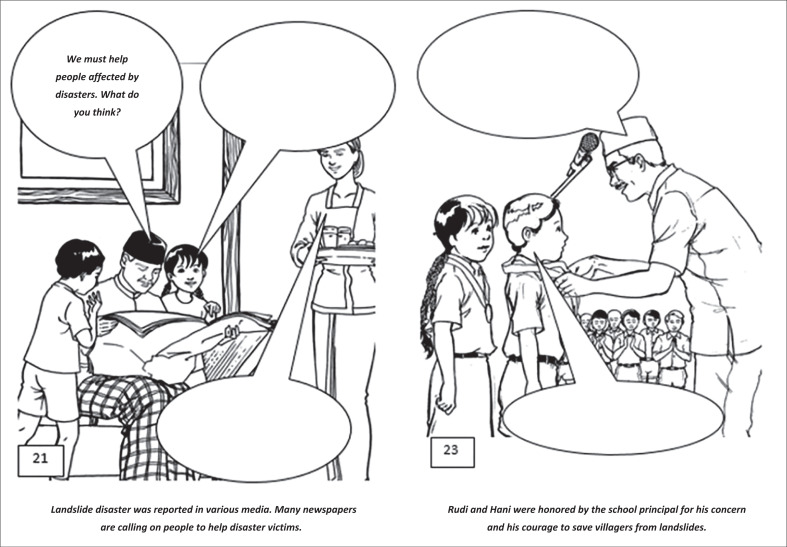
Excerpts from the comic (scenes 21 and 23).

For example, scene 21 depicts an intimate atmosphere in Rudi and Hani’s family while they are listening to their father reading the news on a landslide disaster (Figure 1). The father in the comic then asks Rudi and Hani’s opinion about the plan to help the victims of the landslide disaster. He does not command Rudi and Hani to help the victims but asks the opinions of both the children. This action is the implementation of democratic attitudes. Scene 23 shows the principal presenting an award to Rudi and Hani during the school’s flag-raising ceremony to respond to disaster mitigation in their village (Figure 1).

The two scenes in the picture are a series of stories after the comic characters (Rudi and Hani) manage to save the villagers from the threat of a landslide. The events of the natural disaster were covered in various local newspapers (Scene 21). In the school environment also, there were lot of talks about Rudi and Hani’s desire for helping the villagers, as the principal gave a star of service to Rudi and Hani. The award ceremony was held in front of the learners in order to show them an example of good behaviour (Scene 23).

**FIGURE 2:**
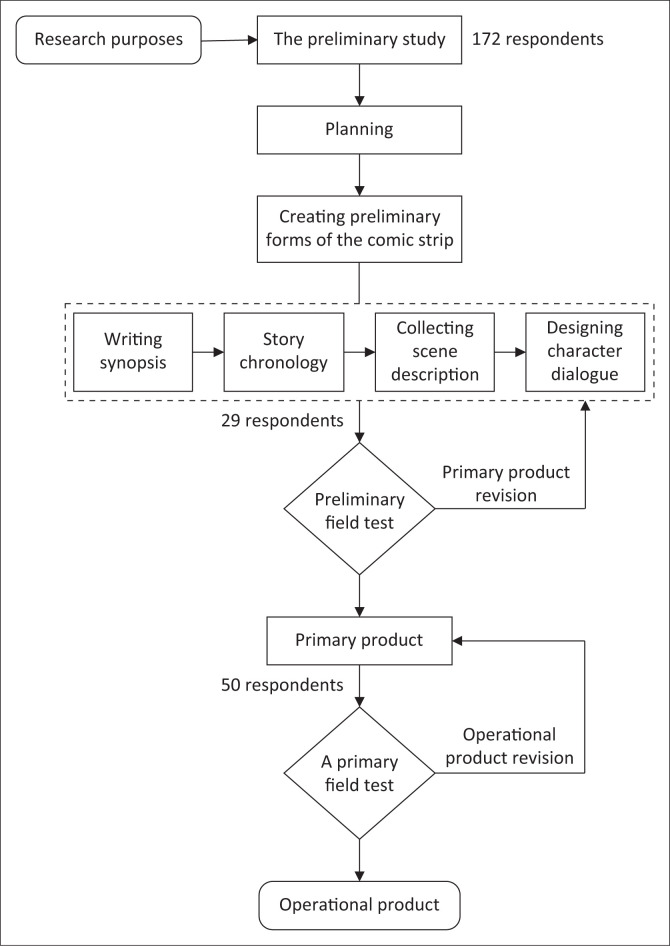
Diagram of the development process in creating comic worksheets.

These comic worksheets have gone through two R&D stages, the preliminary study and model development (see Figure 2).

The preliminary study stage was conducted in 2015 for two and half months. A total of 172 students from two high schools in Bandung, West Java, Indonesia, were asked to fill out a self-administrative questionnaire. The questionnaire consisted of 10 multiple-choice questions about their perceptions of the need for a worksheet with more visualisation using four scale answers (4 = Strongly Agree; 3 = Agree; 2 = Disagree; 1 = Strongly Disagree). The questions were categorised into four primary topics: the benefit of using worksheets in learning activities; advantages and disadvantages of commonly used worksheets; a preference on the comic worksheets; interesting points on reading comics. The result shows that the students’ preference for the comics form worksheets was higher (54%) than the other worksheets (46%). They also argue that comics were more readable than the usual textbook (94%) and increase imagination (83%).

The next stage was model development, which consisted of six steps: (1) Planning, where the researcher compiled the storyboard by writing synopsis and story chronology, collecting scene description, and designing character dialogue; (2) Creating preliminary forms of the product by collaborating with a comics drawing expert; (3) Assessing the comic readability by asking 29 students from two high schools in Bandung to give their opinions and suggestions. The test was also carried out by asking for comics experts’ opinions; (4) Revising the primary product based on the preliminary field test result by increasing the size of comic images and dialogue balloons. The storyline was also clarified by adding information at the bottom of the image; (5) Conducting a primary field test by involving 50 students from five different high schools in Bandung to fill in the comic worksheets; (6) Revising the operational product focusing on the manual instruction for teacher and the image colours. Finally, this study investigated the effectiveness of comic worksheets for operational field testing and explored their values from the obtained data for improvement in the final product revision.

## Research method

The study involved 103 students in total from X grade/16 years old (48 students) and XI grade/17 years old (55 students) in four different high schools in Indonesia. The researcher developed a paper test which consisted of 15 multiple-choice questions and used them for a pre- and post-test. The questions involved knowledge of why disasters occurred, how to deal with them, and what to do when they came. The pre-test was given to students at the end of the geography learning session, focusing on DRR. Afterward, the teacher gave each student a comic worksheet to be taken home and work on. A week later, the completed worksheets were collected again by the teacher, and the students took the post-test. The pre- and post-test design was used to monitor the effects of the comic worksheets on high school students’ conceptual framework development regarding Disaster Risk Mitigation in Geography subject. A paired sample *t*-test was used to analyse the mean difference between the students’ pre- and post-test scores using Statistical Package for the Social Sciences (SPSS) version 20. The N-gain score was also performed when the paired sample *t*-test results showed a significant difference between the two means.

## Findings and discussion

The students had to complete 15 questions on both pre- and post-tests. They were given marks with a maximum score of 15. The pre- and post-test scores showed an overall assessment of the students’ understanding of the landslide’s disasters. The number of questions on each topic are detailed in Table 1.

**TABLE 1:** Topics of questions.

Questions’ topic	Category	Number of questions
Weather conditions	Landslides pre-disaster conditions	1
The process of sowing hygroscopic particles (NaCl) on artificial rain activities	Landslides pre-disaster conditions	3
The process of splash erosion and sheet erosion	Landslides pre-disaster conditions	2
The impact of soil erosion	Landslides pre-disaster conditions	2
Factors and characteristics of land prone to landslides	Disaster mitigation	2
Efforts to prevent landslides	Disaster mitigation	1
The function of an early warning system in areas prone to natural disasters	Disaster mitigation	3
Procedures for victims of landslides	Evacuation	1
Total number of questions		15

NaCl, sodium chloride, also known as salt, or halite.

**FIGURE 3:**
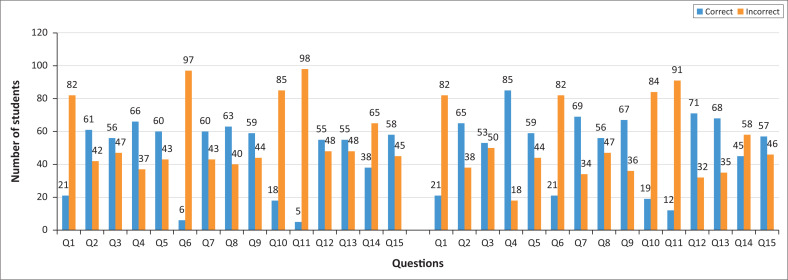
Pre- and post-test results by questions.

The score ranged from 2 to 11 out of 15 for the pre-test, and 3 to 12 out of 15 for the post-test in all classes. Although three students received a very low mark (3 out of 15) both in pre- and post-test, the number of students who received a satisfactory mark (10–12) increased from only 7.8% in the pre-test to 18.4% in the post-test. Question 11 received the lowest number of correct answers (Figure 3). Only 5 out of 103 students (4.8%) chose the correct answers in the pre-test and 12 students (11.6%) in the post-test.

In answering Question 11, regarding the options of efforts to prevent landslides, the students tended to answer the option of reforestation in open land both in hills and lowlands (64.1% in the pre-test and decreasing to 59.2% in the post-test), followed by not to build houses near cliffs (28% both in pre- and post-test), with only 3.1% in pre-test and decreasing to 1.2% in the post-test choosing the option of striving for the sloping cliff to become steady.

Many students did not choose the correct answer (NOT making ponds or plantations on slopes near settlement) because they tended to follow the general framework taught in geography classes where the study of landslides was always associated with deforestation and failure of reforestation. As in Question 11, most students chose an answer whose sentence began with the word ‘reforestation’. This answer was not the correct one because ‘reforestation’ in the conviction was general, explained by the following sentence ‘in the hills and the lowlands’. Areas with low land topography do not have the threat of landslides because they do not have a land slope variable. This finding informs us on how to improve their understanding of the contents through comic worksheets by making the story of the landslides process clearer so that students can better understand the soil layer load because of rainwater infiltration.

**FIGURE 4:**
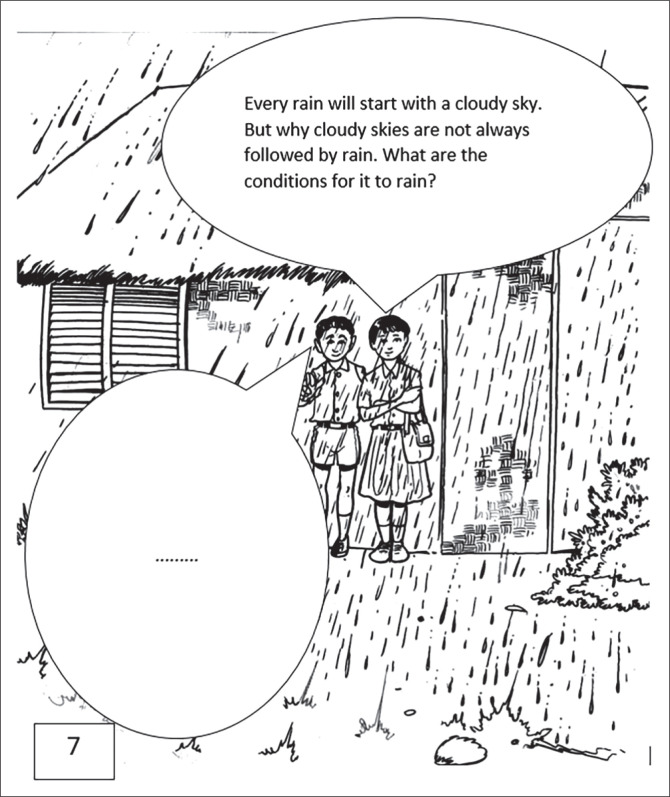
Sample of question in comic worksheets.

In addition to Question 11, four questions (Q1, Q6, Q10, and Q14) received low numbers of correct answers both in the pre- and post-test (see Figure 3). Results for Questions 6, 8, and 14 in the post-test were slightly better than the pre-test. However, the number of correct answers to Question 1, which reads ‘Cloudy weather do not always rain, because the occurrence of rain must meet the requirements, namely, among others is…’ did not increase. This question appeared in the comic (Figure 4), but many students answered only about the condensation process and did not explain the requirements for wind speed and water grain weight, which was the correct answer.

The means across all four classes varied, with the 10th grade of Social Science class from Denpasar High School having the highest mean in pre- and post-test. The 10th-grade students had not yet learned the material about disaster mitigation. They only learned about the process of landslides on the lithosphere chapter. This material was taught in 11th grade. Another interesting result is that the 11th grade Social Science class from Metro-Lampung High School showed a significant increase from the lowest in the pre-test (5.96) to the second highest (7.53) in the post-test. Descriptive statistics for the mean of pre- and post-test for all students (*n* = 103) are summarised in Table 2.

**TABLE 2:** Summary of pre- and post-test means.

High School	Class	Students	Pre-test	Post-test
Denpasar	X Social science	27	7.44	7.70
Sukabumi	X Social science	23	6.39	7.22
Semarang	XI Natural science	25	6.64	7.32
Metro-Lampung	XI Social science	28	5.96	7.53
**Total**		**103**	**6.61**	**7.46**

Table 2 shows the differences between pre- and post-test means among four classes from four different high schools. Post-test means are greater than pre-test for all classes and also for total means. A paired sample *t*-test was calculated to determine if the difference between the tests was significant (Table 3).

**TABLE 3:** Comparison of means between pre- and post-test result (paired sample *t*-test).

Paired samples test	Mean	Std. deviation	Std. error mean	*t*	*df*	Sig. (2-tailed)
Pre-test – Post-test	−0.845	2.062	0.203	−4.158	102	0.000

Std., standard; *t*, t-test; *df*, degrees of freedom; Sig., significant.

The result as shown in Table 3 confirms that there is a significant difference between the pre- and post-test. The minimum N-Gain value was -150%, and the maximum was 100%. The N-Gain score was 8.87%, which was >76%, implying that comic worksheets effectively helped high school students develop their conceptual framework about DRR (Table 4).

**TABLE 4:** N-Gain score calculation.

Parameters	Statistic	Std. error
**N_Gain_score**	8.8716	4.25108
Mean	0.4396	-
95% confidence interval for mean	17.3036	-
Lower bound	-	-
Upper bound	-	-
5% Trimmed mean	11.4699	-
Median	14.2857	-
Variance	1861.384	-
Std. deviation	43.14376	-
Minimum	−150.00	-
Maximum	100.00	-
Range	250.00	-
Interquartile range	33.33	-
Skewness	−1.026	0.238
Kurtosis	2.302	0.472

Std., standard.

**FIGURE 5:**
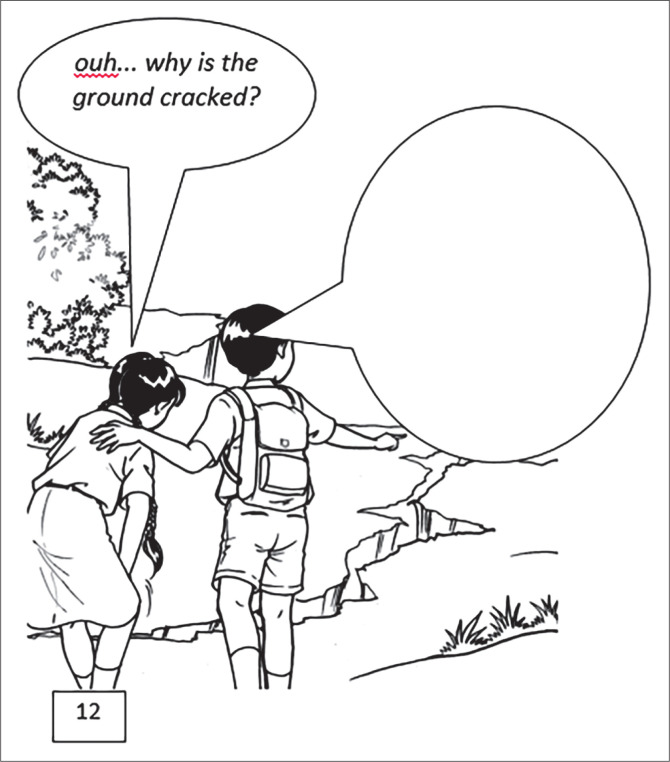
Scene 12 is designed to evoke skills in arguing for cracked soils as the initial indicator of landslide activity.

The scenes in the comic worksheets containing the problems in reducing disaster are arranged with reference to four characteristics of questions in inquiry learning. First, the questions are open-ended, allowing more than one correct answers. Second, questions require answers with higher-order thinking skills including analysing, concluding, reflecting, and evaluating. Third, questions need support and reasons. Fourth, answers often lead to debate, discussion, and new questions (Gholam 2019). Students have the opportunity to submit their opinions based on their problem identification, analysis, and conclusions as mediated by comics. All students are involved in expressing their opinions in solving problems or answering questions. This is reflected in the variety of arguments written by students in Scene 12 (Figure 5), which is about cracked soil as the initial indicator of landslide activity.

The following is an example of an argument about the initial indicator of landslide activity in the comic worksheet, scene 12:

1. Aurelia (from Denpasar): ‘It was because of the long dry season, then it rained. It causes the soil to crack’.
2. Dwina (from Sukabumi): ‘You remember, it’s called hoof cracks, one of the natural indicator that a landslide will occur. Save life’.
3. Arda (from Metro-Lampung): ‘… During the rainy seasons, rainwater infiltrates into the cracked soils, so that the soil shifts. The shifting soil causes erosion and then landslides occur’.
4. Ainnur (from Semarang): ‘Because rainwater flows and erodes the soil surface, resulting in bigger soil cracks’.
5. Ida Ayu (from Denpasar): ‘Maybe because of the heavy rains’.
6. Bima (from Semarang): ‘It’s due to absence of trees, the soil may crack and that is the first indicator of a landslide’.

Further, in scene 12, the dialog involving such characters is made by involving questions that require higher order thinking skills (HOTS) such as doing an analysis, drawing a conclusion, reflection, and evaluation. The answer requires supporting data for discussion.

**FIGURE 6:**
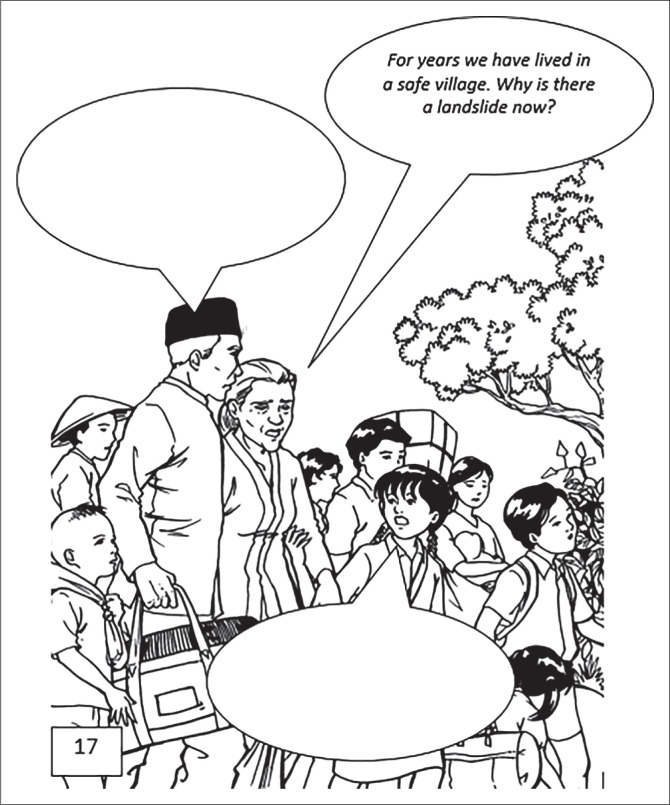
Scene 6 that requires students to analyse the factors that cause landslides.

In Scene 6 (Figure 6), one of the characters asks about the landslide disaster that first occurred in their village, after years of being safe from the threat of disaster. From these questions, students come up with various answers. Some answered that it was caused by heavy rains, the condition of the cliffs being eroded, the condition of the cliffs being fragile, and the hills being bare because of illegal logging (deforestation). The various answers indicate that the comic worksheets have functioned effectively in stimulating students’ critical and argumentative thinking.

The data obtained from the blank speech bubbles filled by the students were also explored to see if particular scenes can be used by teachers to assess students’ character values. Of the 25 scenes, eight scenes were designed for the assessment (Table 5).

**TABLE 5:** Scenes for character values assessment.

Scenes	Character values
1	Saying goodbye to parents when going to school
3	Expressing gratitude
15	Caring and being proactive in disaster mitigation
17	Helping to save residents from disasters
19	Gratitude for surviving the disaster
20	Comforting disaster victims
21	Being democratic
23	Conveying respect for others

In scene 1, all students wrote, ‘I will leave first’ as the common expression of saying goodbye to their parents when going to school. Some Muslim students added the word ‘*Assalamualaikum*’ a greeting in Arabic which means ‘peace be upon you’. Only a few of them added a request for prayer to their mother so that he or she could study well at school. Students’ writings for the mother’s answer were varied, such as advising on being careful on the school trip, reminding them of having a home-made meal and protecting each other. However, there was one student who wrote the dialogue with offensive and disrespectful language. The students were aware that the teacher would read their writing. Thus, they may be providing the answers to get their teacher’s attention, so the teacher would respond to them.

In scene 3, all students wrote ‘thank you, mom’ as the expression of gratitude to their mother who reminded her children to bring umbrellas. A few of them added apologies for forgetting an essential item. Scenes 15 and 17 describe the most critical behaviour in disaster mitigation. The scene was deliberately designed to show attitudes of caring and working proactively in facing the threat of natural disasters. Informing other people about the signs of a landslide is not easy. It needs communication skills for the information to be delivered, understood, and trusted by the recipients (communicants). Most students have already provided the correct information and were able to decide what to do. Although there were still a few students who wrote that there was a sign of landslides without writing what the sign was, many had written the information about ground cracks as a sign.

Scenes 19 and 20 explore the reactions of students in facing disasters. Some of them chose the theme of dialogue in dealing with the aftermath of a disaster, but some decided to have conversations on moral and religious issues, such as remaining grateful and be patient in facing the test of God and encourage others to be resilient. Scene 21 shows a dialogue between the parents and the children. The father asks the children’s opinions about what to do to help disaster victims. This scene gives the students an example of the democratic attitude of the father by asking the children to think and talk about their opinions. Almost all students answered to donate money, food, clothing, and medicine to help the victims, while others answered to raise funds for the victims.

Scene 23 is a scene where the school principal gives the children an award for saving the villagers from the threat of landslides. Through this scene, students are invited to think about how to give and receive rewards from others. All students wrote only ‘thank you’, but one student added her hope for the future by writing ‘thank you, sir, I hope this award will also motivate other students to do the same in the future’.

The data described above shows the creativity of students in expressing their ideas in writing. The written language used varies in expressing the same idea. Although written language does not always reflect students’ actual attitudes or behaviours, their decision to choose a positive or negative language shows their attitude towards what is considered acceptable. Students’ dialogue can be regarded as a recording of their attitudes and behaviours in their everyday life. When teachers find it difficult to assess student attitudes through interviews or daily observations in the classroom, this kind of comic worksheets can be used as a portfolio to help them do the attitude assessment.

## Conclusion

Integrating DRR material in Geography is a common practice but by no means without obstacles. Knowledge-orientation of the subjects is often considered less in line with the objectives of DRR to develop students’ competencies and skills so that they are able to contribute proactively to disaster prevention and mitigation. The researcher has tried to harmonise the two learning points in comic worksheets to guide students in learning both materials. Although several previous studies have shown the ineffectiveness of worksheets in helping students learn, those studies did not take into account how the worksheets should be designed, developed, and used. This comic worksheets were designed and developed through the R&D stages. To improve the final product revision, the effectiveness of the comic worksheets was tested. The obtained data were also explored to see its ability to assess the students’ attitude assessment. The paired sample *t*-test result confirmed a significant difference between the pre- and post-test and the N-Gain score (8.87%), implying that the comic worksheets were effective in assisting high school students in learning DRR in Geography subjects. Teachers can use these worksheets as a portfolio to document students’ cognitive and affective aspects simultaneously. Several scenes are directed at cognitive aspects and have an instructional effect.

Meanwhile, others are directed towards affective aspects to give a nurturant effect. However, developing such worksheets requires many parties, such as teachers, instructional media developers, artists, or even layout designers, if produced in a book form. The implication of this study relates to the need for collaborative teamwork with an adequate budget.
